# Interaction between NMDA Receptor- and Endocannabinoid-Mediated Modulation of Nociceptive Synapses

**DOI:** 10.1038/s41598-018-37890-z

**Published:** 2019-02-04

**Authors:** Sharleen Yuan, Brian D. Burrell

**Affiliations:** Center for Brain and Behavior Research, Division of Basic Biomedical Sciences Sanford School of Medicine, University of South Dakota, Vermillion, SD 57069 USA

## Abstract

Nociceptors, sensory neurons that detect damage or potential damage to the body, are the first stage of communicating noxious stimuli from the periphery to central nervous system (CNS). In this study, long-term potentiation (LTP) in the CNS of the medicinal leech, *Hirudo verbana*, was examined, taking advantage of the ability to selectively record from nociceptive synapses in this model organism. High frequency stimulation (HFS) of nociceptors produced a persistent increase in synaptic transmission and this LTP was both NMDA receptor-mediated and synapse-specific. Surprisingly, inhibition of NMDA receptors during HFS “uncovered” a persistent form of depression. This long-term depression (LTD) was mediated by the endocannabinoid 2-arachidonoyl glycerol (2-AG) acting on a TRPV (transient receptor potential vanilloid) –like channel. These observations suggest that (1) NMDA receptor mediated LTP is observed in nociceptors across both vertebrate and invertebrate phyla and (2) there may be an interaction between NMDA receptor-mediated and endocannabinoid-mediated forms of synaptic plasticity in nociceptors. Specifically, the NMDA receptor mediated processes may suppress endocannabinoid signaling. Such findings could be significant for understanding cellular mechanisms behind nociceptive sensitization and perhaps their contribution to chronic pain.

## Introduction

Nociceptors are somatosensory neurons dedicated to detecting damaging or potentially damaging stimuli, which then transmit that information to the rest of the central nervous system (CNS)^1^. In both invertebrates and vertebrates, nociceptors are distinct from somatosensory neurons that detect and transmit innocuous mechanical and thermal stimuli^2–4^. Activation of nociceptors can elicit motor behaviors designed to withdraw or escape from the noxious stimuli^2,5–7^, but these afferents also activate modulatory circuits that help the animal adapt in its encounter with these stimuli^8–10^. Consequently, changes in the strength of synaptic transmission by nociceptors represent a critical element in how animals respond to future encounters with noxious stimuli. For example, potentiation of nociceptive synapses can contribute to behavioral sensitization that serves to protect the animal from further damage^10^. Elucidating the cellular mechanisms that mediate potentiation of nociceptive synapses will contribute to our understanding of how animals make adaptive changes following injury. Such information could also be applied to develop new therapeutic approaches in treating pathological forms of nociceptive sensitization in humans, i.e. chronic pain.

Long-term potentiation (LTP) is a form of activity-dependent synaptic strengthening observed practically throughout the CNS and has been documented in vertebrate^11^ and invertebrate species^12–14^. It is a homosynaptic form of synaptic plasticity and is often mediated by NMDA receptors (NMDARs) that require both glutamate-binding and postsynaptic depolarization in order to become active. One can envision that injury-inducing stimuli could activate nociceptors enough to elicit LTP in nociceptive synapses in the spinal cord and, in fact, NMDAR-mediated LTP (NMDAR-LTP) has been observed in rodent C fiber synapses following repetitive stimulation^11,15,16^. However, studying LTP in nociceptive synapses in the spinal cord and its contribution to nociceptive sensitization is complicated by a variety of factors. These include the perhaps surprising complexity of the nociceptive and non-nociceptive circuitry in the spinal cord^17,18^, the fact that different patterns of stimulation are required for LTP in different nociceptive pathways^11,15,16^ and questions as to whether NMDAR-LTP in spinal nociceptive synapses exhibit synapse specificity similar to what is observed in other regions of the CNS^19^.

The CNS of *Hirudo verbana* (the medicinal leech) provides a tractable system to examine LTP in nociceptive synapses. The *Hirudo* CNS is organized as a chain of ganglia that runs the length of the animal with each ganglion having its own complement of touch- and pressure sensitive neurons as well as bilateral pairs of mechano-sensitive and polymodal nociceptive neurons^20–23^. *Hirudo* nociceptive sensory neurons (N cells) have glutamatergic synaptic input to motor neurons and interneurons responsible for reflexive withdrawal and locomotory escape behaviors^6,24,25^. These N cells also have input onto serotonergic cells that play a critical role in modulating *Hirudo* behaviors in the context of learning and responses to stress and hunger^26–29^. *Hirudo* pressure (P) cell and touch (T) cell synapses do undergo LTP that is NMDAR-dependent and synapse specific^14,30,31^, but LTP in the leech N cell synapses has not been investigated. In this study we examined the capacity of *Hirudo* N synapses to undergo NMDAR-LTP. We found evidence supporting the presence of LTP in these nociceptive synapses that is NMDAR-dependent and synapse-specific, but also found, to our surprise, evidence of an interaction between NMDAR-mediated synaptic potentiation and endocannabinoid-mediated synaptic depression.

## Materials and Methods

*Hirudo verbana* (3g) were obtained from commercial suppliers (Leeches USA, Westbury, NY and Niagara Leeches, Niagara, NY) and maintained in artificial pond water [0.52 g acquarium salt (Instant Ocean) per liter of H_2_O] on a 12 hour light/dark cycle at 15 °C. Individual ganglia were dissected and pinned in a recording chamber with constant perfusion of normal *Hirudo* saline (110 mM NaCl, 5 mM NaOH, 4 mM KCl, 1.8 mM CaCl_2_, 1 mM MgCl_2_, and 10 mM HEPES, pH = 7.4) at an approximately 1.5 mL/min.

**Figure 1:**
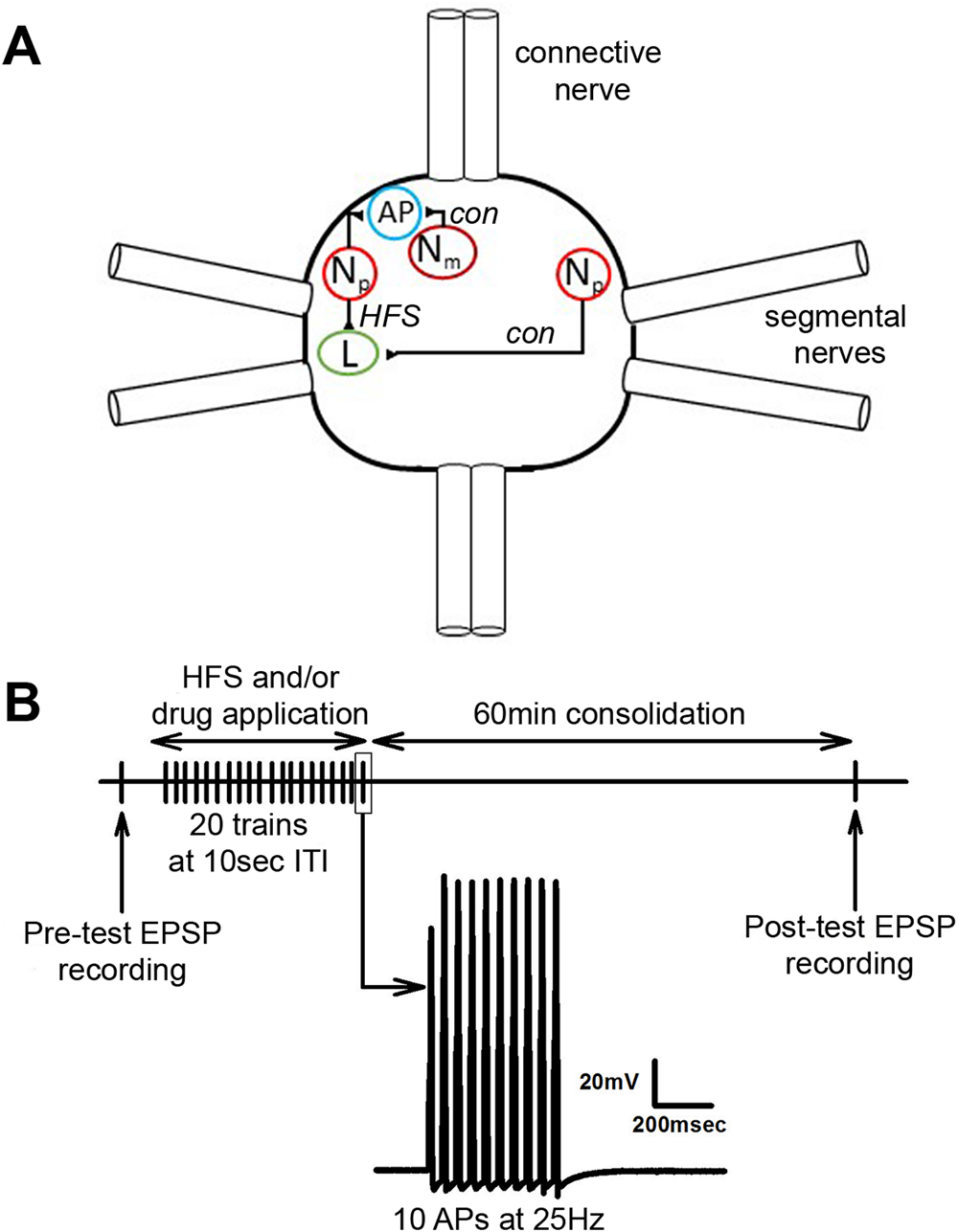
(**A**) Circuit diagram of neurons recorded in this study. A single body ganglion (1 of 21) is shown. Each ganglion has two pairs of segmental nerves that project to the periphery and a connective nerve that links each ganglion to its anterior and posterior neighbors. All of the neurons shown are actually bilateral pairs, but the contralateral cells for all but the N_P_ have been omitted in this figure for the sake of clarity. In all LTP experiments, HFS was delivered to the lateral N cell (also known as the polymodal N or N_p_). To test synapse specificity of LTP between bilateral pairs of N_p_ cells, changes in synaptic transmission was compared between the ipsilateral N and the contralateral N signaling onto the L motor neuron. To test synapse specificity of LTP between ipsilateral pairs of N cells, changes in synaptic transmission was compared between the lateral N_P_ and the medial N (also known as the mechanical N cell or N_m_) signaling onto the AP neuron. (**B**) Experimental protocol. Following a pre-test of the N cell EPSP, HFS was applied (see Methods) followed by a 60 min consolidation period and then a post-test measurement of the same N cell synapse.

Individual neurons were identified based on their position within the ganglion, size, and electrophysiological properties. Each ganglion contains two bilateral pairs of nociceptive (N) and pressure-sensitive cells and three pairs of light touch sensitive neurons. The N cells are further divided into a lateral N pair that are polymodal nociceptors (N_poly_) and a medial pair that are mechanical nociceptors (N_mech_; Fig. 1A)^21–23^. One postsynaptic target of the N cells is the longitudinal (L) motor neuron which contributes to the defensive withdrawal reflex elicited by noxious stimulation^32^. L motor neuron identification was confirmed by recording from the electrically coupled contralateral L motor neuron and observe synchronous activity^33^. The L cells are located on the dorsal side of the ganglion and the afferent cells are located on the ventral surface. However, it is possible to record from both the L and the N_poly_ or P cells from the dorsal side of the ganglion given the lateral positions of both these primary afferents. It is not possible to record from the N_mech_ cell when the ganglion is dorsal side up because this neuron is located medially on the ventral side of the ganglion. Therefore, synaptic recordings were made from the N_mech_ and anterior pagoda (AP) neurons which also receive input from both types of N cells (Fig. 1A) and are all located on the ventral side of the ganglion. Where they have been compared, the properties of N- or P-to-AP synapses appear to be identical to other synaptic connections made by these sensory cells^34,35^.

Drugs used for each experiment were kept as frozen aliquot solutions and then diluted to their final concentration in normal saline just before respective experiment. SB366791 (SB) and tetrahydrolipstatin (THL, also known as Orlistat) were obtained from Tocris/Bio-Techne (Minneapolis, MN) and stocks were made in dimethyl sulfoxide (DMSO). DL-2-Amino-5-phosphonopentanoic acid (AP5; stocks made in normal saline), 1,2-bis(*o*-aminophenoxy)ethane-*N*,*N*,*N′*,*N′*-tetraacetic acid (BAPTA), and DMSO were obtained from Sigma-Aldrich (St. Louis, MO).

Electrophysiological recording techniques have been described in detailed previously^36^. Briefly, current clamp (bridge balanced) intracellular recordings were carried out using sharp glass microelectrodes (tip resistance 20–35 MΩ) made from borosilicate capillary tubing (1.0 mm OD, 0.75 mm ID; FHC, Bowdoinham, ME) using a horizontal puller (Sutter Instruments P-97; Novato, CA). Microelectrodes were filled with 3 M potassium acetate (KAc). Manual micropositioners (Model 1480; Siskiyou Inc., Grants Pass, OR) were used to impale individual neurons during experiments. Current pulses were delivered to electrodes using a STG 1004 Multi-Channel Systems programmable stimulator (Reutlingen, Germany). Membrane potential data were recorded using a bridge amplifier (BA-1S; NPI, Tamm, Germany) and digitally converted for analysis using a Digidata 1322 A (Molecular Devices, Sunnyvale, CA).

High-frequency stimulation (HFS) of N_poly_ consisted of 20 trains, 10 action potentials per train at 25 Hz, and a 10 s inter-train interval. EPSP and IR recordings were made prior to (pretest) and 60 min following HFS (Fig. 1B). This procedure has been used in previous experiments to elicit homosynaptic and heterosynaptic forms of LTP^14,37^. In experiments using BAPTA, 1 mM BAPTA was included in the electrode filling solution and the Ca^2+^ chelator was iontophoretically injected into the L motor neuron (-1nA holding current for 5 minutes) prior to HFS. For all experiments, electrodes were withdrawn following pre-test recordings and the neurons re-impaled for the post-test recordings approximately 1 hr later. This was done, in part, because prolonged recordings with these sharp microelectrodes results in rundown of EPSP amplitudes likely due to damage to the recorded cell. To insure that changes in synaptic transmission were not the result of changes in post-synaptic input resistance (IR), this was monitored during the pre- and post-test recordings by measuring the membrane potential change during a 500 msec, 1 nA negative current pulse. Only stable recordings (<10% change in IR) were included in the final data analysis. During the pre- and post-tests, EPSPs were elicited at 0.1 Hz and the peak amplitude was calculated from the average of 5–10 EPSPs. EPSP amplitude measurements of the pre- and post-test recordings were normalized and presented as the mean ± SE. Statistical analyses using t-test and one-way analysis of variance (ANOVA) were performed to determine main effects with Student-Newman-Keuls post hoc tests to confirm the ANOVA results. All analyses were carried out with Sigmaplot. All significance was determined at α level of at least *P* ≤ 0.05.

## Results

**Figure 2:**
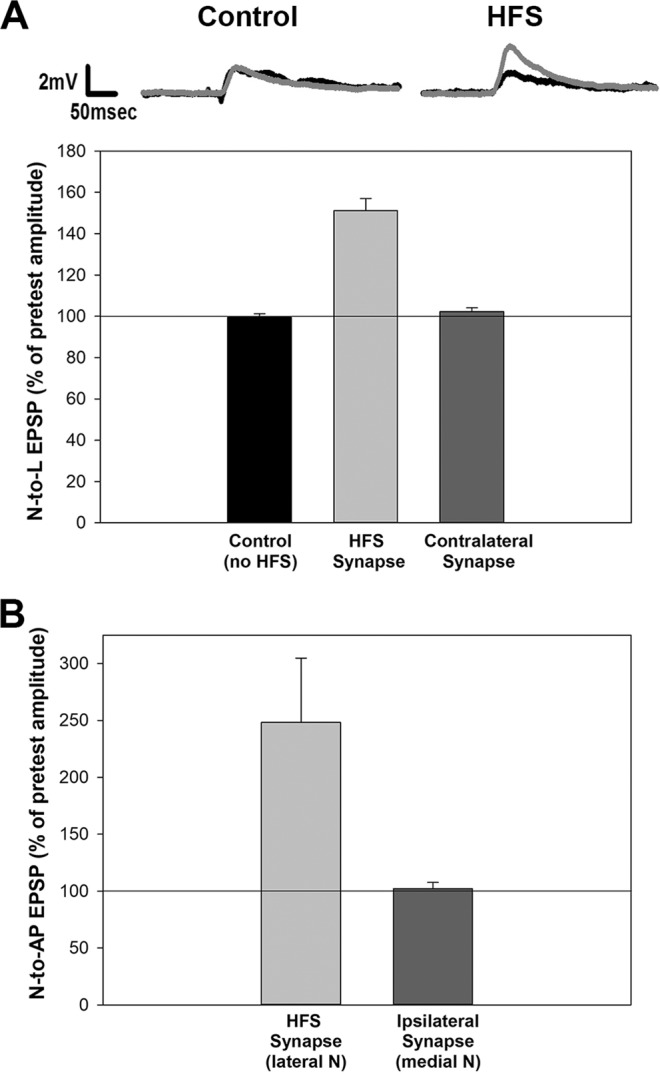
Synapse specificity of LTP in *Hirudo* nociceptive synapses. (**A**) HFS elicited substantial potentiation in the N-to-L synapse that underwent LFS (HFS synapse), but no change in EPSP size was observed in the synapse made by the contralateral N cell onto the same postsynaptic target (Contralateral synapse). No change in the N-to-L EPSP was observed in experiments in which the HFS was omitted (Control). (**B**) In experiments in which both N cells were ipsilateral to each other, HFS of the lateral N cell elicited potentiation in that nociceptor’s synapses, but no potentiation was observed in the synapse made by the ipsilateral N cell (the N_m_) onto the same postsynaptic target.

The first experiments were to assess whether HFS of a single nociceptor could elicit LTP that was synapse specific. L motor neurons receive synaptic input from both contralateral and ipsilateral afferents and the morphology of both the motor neuron and sensory cell arborizations permits synaptic contacts on both sides of the ganglion^24,38^. HFS of a N_poly_ cell did result in substantial potentiation of the EPSP elicited in the ipsilateral L motor neuron recorded 1 hr later (Fig. 2A; ANOVA F_2,14_ = 60.95; *post-hoc* HFS vs. control p < 0.001). No potentiation was observed in the synapse made by contralateral N_poly_ onto the same postsynaptic L cell (Figs 1A and 2A; *post-hoc* p > 0.05). A separate set of experiments was carried to examine whether HFS of one nociceptor would influence synaptic transmission by an ipsilateral nociceptive afferent onto the same postsynaptic target. Specifically, these effects were examined in the N_poly_ and N_mech_ synapses onto a shared AP neuron that was ipsilateral relative to both nociceptors (Fig. 1A). Again, HFS of the lateral N_poly_ produced significant potentiation 1 hr later compared to the medial N_mech_, in which no potentiation was observed (Fig. 2B; t_1,8_ = 2.58, p < 0.05). Together these results demonstrate that HFS on a single nociceptor elicits homosynaptic LTP that is synapse specific.

**Figure 3:**
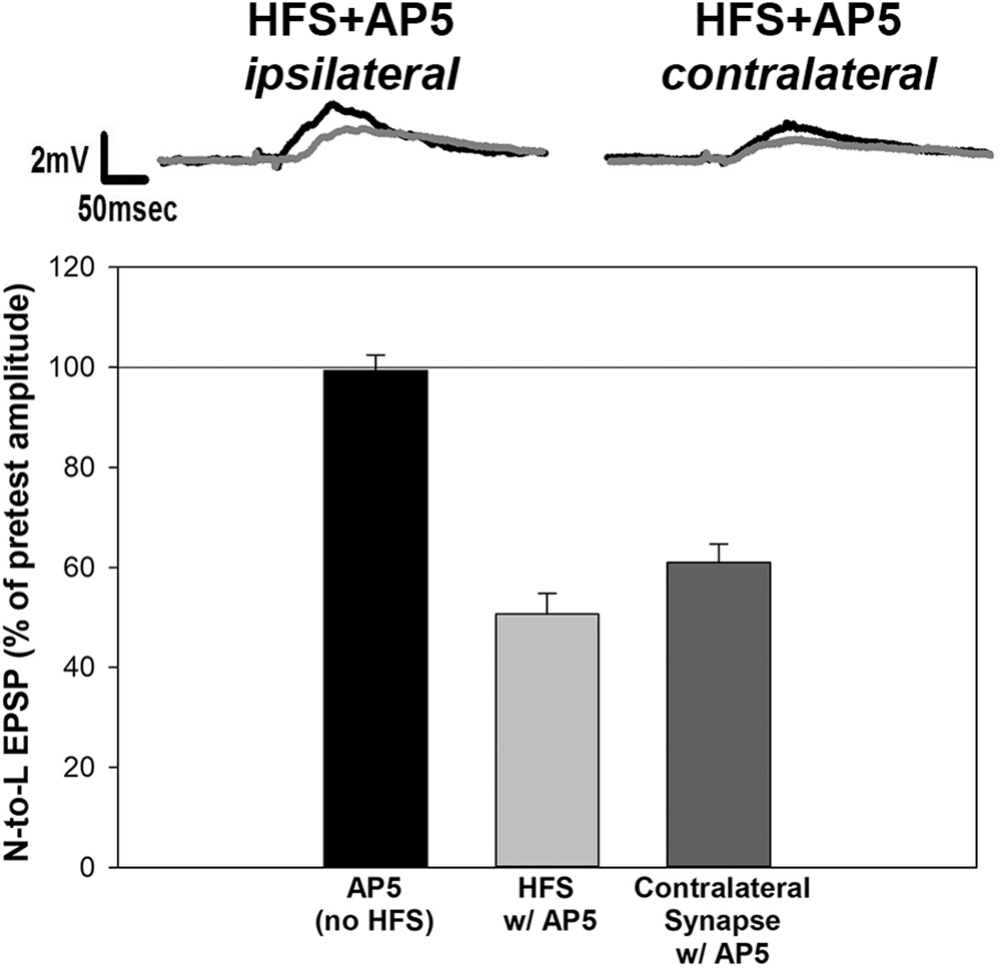
LTD was observed in the HFS + AP5 conditions. Experiments were repeated in which synaptic transmission by contralateral N cells onto a shared postsynaptic target were measured (see Fig. 1A), but in the presence of AP5. In these conditions, LTD was observed in the N cell synapse that received HFS. LTD was also observed in the contralateral synapse that did not receive HFS. AP5 alone does not alter EPSP magnitude in these experiments.

Next, the potential role of NMDARs during N-cell LTP was examined. HFS was delivered in the presence of 100 µM AP5 which has been shown to block LTP in *Hirudo* and *Aplysia* synapses^13,14,31,39^. AP5 delivered alone (no HFS) had no persistent effect on the nociceptive synapses (Fig. 3). No LTP was observed in N-to-L synapses that received HFS in the presence of AP5. Surprisingly, a statistically significant decrease in the N-to-L EPSP was observed in these HFS + AP5 synapses (Fig. 3; F_2,14_ = 58.23, *post-hoc* for AP5 vs. AP5 + HFS p < 0.001). Furthermore, the synapse made by the contralateral N cell onto the same postsynaptic L motor neuron, which was unchanged by HFS in normal saline (Fig. 2A), was depressed in the AP5 + HFS condition (Fig. 3; *post-hoc* for AP5 vs. Contralateral AP5 + HFS p < 0.001). A comparison of the contralateral synapses tested in the AP5 + HFS (Fig. 3) condition versus the contralateral synapses tested in the HFS + saline condition also reveal a statistically significant difference (Fig. 2A; t = 9.76, p < 0.0001). These finding indicate that (1) LTP in the N-to-L synapse is mediated by NMDARs, (2) blocking NMDAR activity during HFS uncovers a form of long-term depression (LTD) in the activated synapse and (3) this same HFS produced heterosynaptic LTD in the synapses made by the contralateral N cell onto the same postsynaptic target.

**Figure 4:**
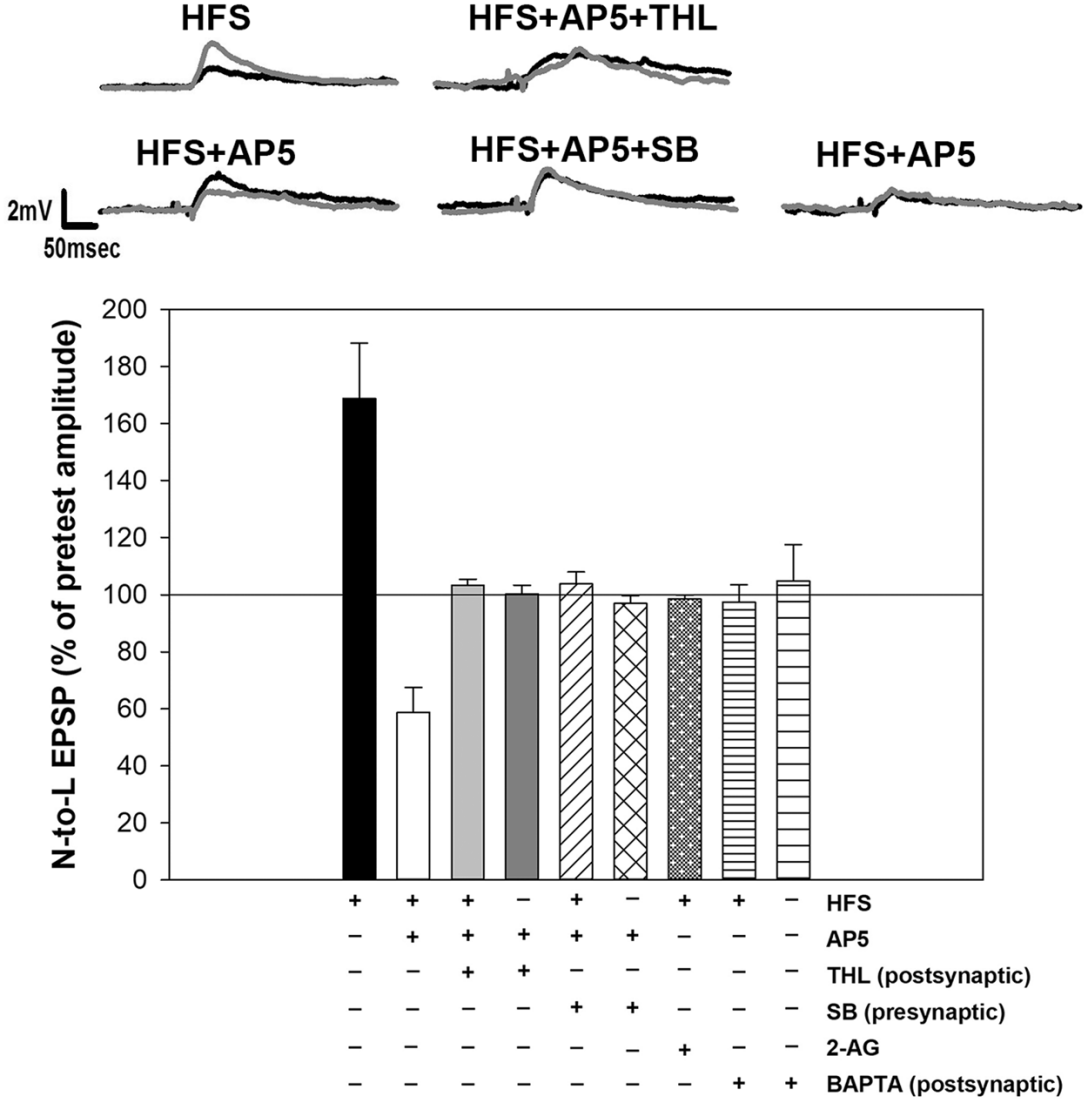
LTD observed in the HFS + AP5 conditions require endocannabinoid signaling. LTD was blocked in experiments where the HFS + AP5 treatment also included either injection of THL into the L motor neuron (HFS + AP5 + THL) or injection of SB366791 into the N cell (HFS + AP5 + SB). In experiments where HFS was omitted, neither AP5 + THL nor AP5 + SB produce any change in the N-to-L EPSP. In experiments in which HFS was delivered in the presence of 2-AG, no potentiation was observed, indicating that N cell synapses that received HFS were still sensitive to 2-AG. Postsynaptic injection of BAPTA prior to HFS prevented both LTP and LTD of the nociceptive synapses.

What mechanism might explain the appearance of LTD in synapses that receive HFS in the presence of a NMDAR antagonist? Low frequency stimulation (LFS) of afferents with input onto the L motor neuron elicits LTD in N-to-L synapses that is heterosynaptic and mediated by endocannabinoid signaling. Specifically, LFS (1 Hz stimulation for 15 mins) elicits synthesis of the endocannabinoid 2-arachidonoyl glycerol (2-AG) in the postsynaptic L motor neuron, which then acts in a retrograde manner on TRPV-like channels on the N cell to produce synaptic depression^6,36,40^. Perhaps, blocking NMDAR activity during HFS of afferent-to-L inputs is permissive to a form of 2-AG/TRPV signaling that is normally inhibited by HFS. To test this hypothesis, we first tested the effects of HFS plus AP5 in synapses in which the DAG lipase inhibitor THL (10 µM; DAG lipase is responsible for 2-AG synthesis) was iontophoretically injected into the L motorneuron (HFS + AP + THL). No change in EPSP amplitude was observed in the HFS + AP5 + THL group indicating that the synaptic depression previously observed in the HFS + AP5 condition had been blocked (Fig. 4; F_8,42_ = 10.92, p < 0.001; HFS + AP5 vs. HFS + AP5 + THL *post-hoc* p < 0.01). Injection of THL into the L cell with AP5, but without HFS had no effect on EPSP amplitude.

Next, we examined the role of the TRPV-like receptor. TRPV1 can act as an endocannabinoid receptor in mammals and a TRPV-like channel has been proposed to have a similar function in invertebrates^4,41,42^. In these experiments, we utilized SB366791, a selective inhibitor of mammalian TRPV1 channels that acts as a competitive antagonist at the intracellular capsaicin binding site, but also blocks TRPV1 activation by other stimuli^43^. In *Hirudo*, SB366791 has been shown to block the ability of 2-AG to elicit LTD in N cell synapses and also blocks LFS-elicited depression at these synapses^6,36^. In the current experiments the SB366791was iontophoretically injected into the N cell prior to HFS in ganglia that were also treated with AP5 (HFS + AP5 + SB). No change in EPSP amplitude was observed in the HFS + AP5 + SB group indicating that the synaptic depression previously observed in the HFS + AP5 condition had been blocked as when the presynaptic TRPV-like channel was inhibited (Fig. 4; HFS + AP5 vs. HFS + AP5 + SB *post-hoc* p < 0.01). Injection of SB366791 in the N cell with AP5, but without HFS had no effect on EPSP amplitude.

2-AG normally elicits significant depression in synapses that have not undergone HFS^6,36,40,44^. Therefore, the ability of synapses that had undergone HFS to respond to 2-AG was examined to determine whether these synapses were still sensitive to this endocannabinoid. 100 µM 2-AG was bath-applied to ganglia during HFS and then the EPSP amplitude was tested 60 mins later. In these experiments, neither potentiation nor depression of the EPSP was observed. This suggests that the potentiation induced by the HFS was offset by the depression induced by 2-AG, with the end result being no change in EPSP amplitude.

Finally, the potential role of intracellular Ca^2+^ was examined since increases in intracellular postsynaptic Ca^2+^ are required for both NMDAR-LTP in other *Hirudo* synapses and endocannabinoid-mediated LTD (eCB-LTD) in nociceptive synapses^30,31,44^. In experiments where 1 mM BAPTA was iontophoretically injected into the L motor neuron, HFS failed to elicit any change in the EPSP amplitude (Fig. 4) indicating that there was no LTP (HFS vs. HFS + BAPTA *post-hoc* p < 0.001) nor LTD (HFS + AP5 vs. HFS + BAPTA *post-hoc* p < 0.05). Injection of BAPTA into the L motor neuron without HFS had no effect on EPSP amplitude. These findings suggest that postsynaptic Ca^2+^ signaling is required for both NMDAR-LTP and the eCB-LTD that is uncovered when LTP is blocked.

## Discussion

How activity-driven processes potentiate synaptic transmission by nociceptors is of critical interest since such changes are likely to contribute to behavioral sensitization to nociceptive stimuli^10^. LTP mediated by NMDARs, previously shown to be an important modulator of synaptic signaling in other regions of the CNS, has been considered a potential candidate for contributing to nociceptive sensitization^45,46^. Indeed, NMDAR-LTP has been observed in nociceptive synapses in the mammalian spinal cord, although different nociceptive pathways appear to require different patterns of activity to elicit LTP^15,47^. There has been some question in the mammalian literature as to whether NMDAR-LTP, which is synapse specific in other regions of the CNS, contributes to nociceptive sensitization since this can include both sensitization at the site of injury (primary hyperalgesia) and away from the site of injury (secondary hyperalgesia)^48,49^. The present study using *Hirudo* shows that nociceptive synapses are capable of NMDAR-LTP that is synapse specific. This was observed for both contralateral pairs of nociceptors and ipsilateral pairs that converge onto a common postsynaptic target. Earlier studies in *Hirudo* and in *Aplysia* (a marine mollusk) have also demonstrated NMDAR-mediated LTP in afferent synapses that is synapse specific^14,50,51^.

That synapse-specific NMDAR-LTP occurs in primary nociceptors synapses is not inconsistent with the presence of both primary and secondary nociceptive sensitization in invertebrates or hyperalgesia in mammals. Noxious stimuli that elicit homosynaptic LTP in primary nociceptors can also initiate heterosynaptic modulatory processes that contribute to sensitization^52^. Furthermore, the homosynaptic processes are in a position to contribute to primary (or site-specific) sensitization while heterosynaptic processes can contribute to generalized or secondary sensitization via a wide variety of modulatory mechanisms^10^. In one recent examples of such a process, induction of LTP in rodent C fiber synapses was found to stimulate the release of cytokines and D-serine by astrocytes and/or microglia which produced heterosynaptic potentiation in non-activated nociceptive synapses^16^. Interestingly, in *Hirudo*, the same HFS of N cells that produced NMDAR-LTP in nociceptive synapses also produced a heterosynaptic potentiation in pressure (P) cell synapses^37^. In this situation, potentiation of the P cell synapses was due to endocannabinoid-mediated depression of GABAergic input to P cells, resulting in a persistent form of disinhibition. These findings were replicated in semi-intact preparations in which HFS of N cells in the same segment or noxious stimuli delivered several segments away produced endocannabinoid-mediated potentiation of P cell synapses and sensitization of reflexive withdrawal behaviors elicited by P cell activation^53^.

**Figure 5:**
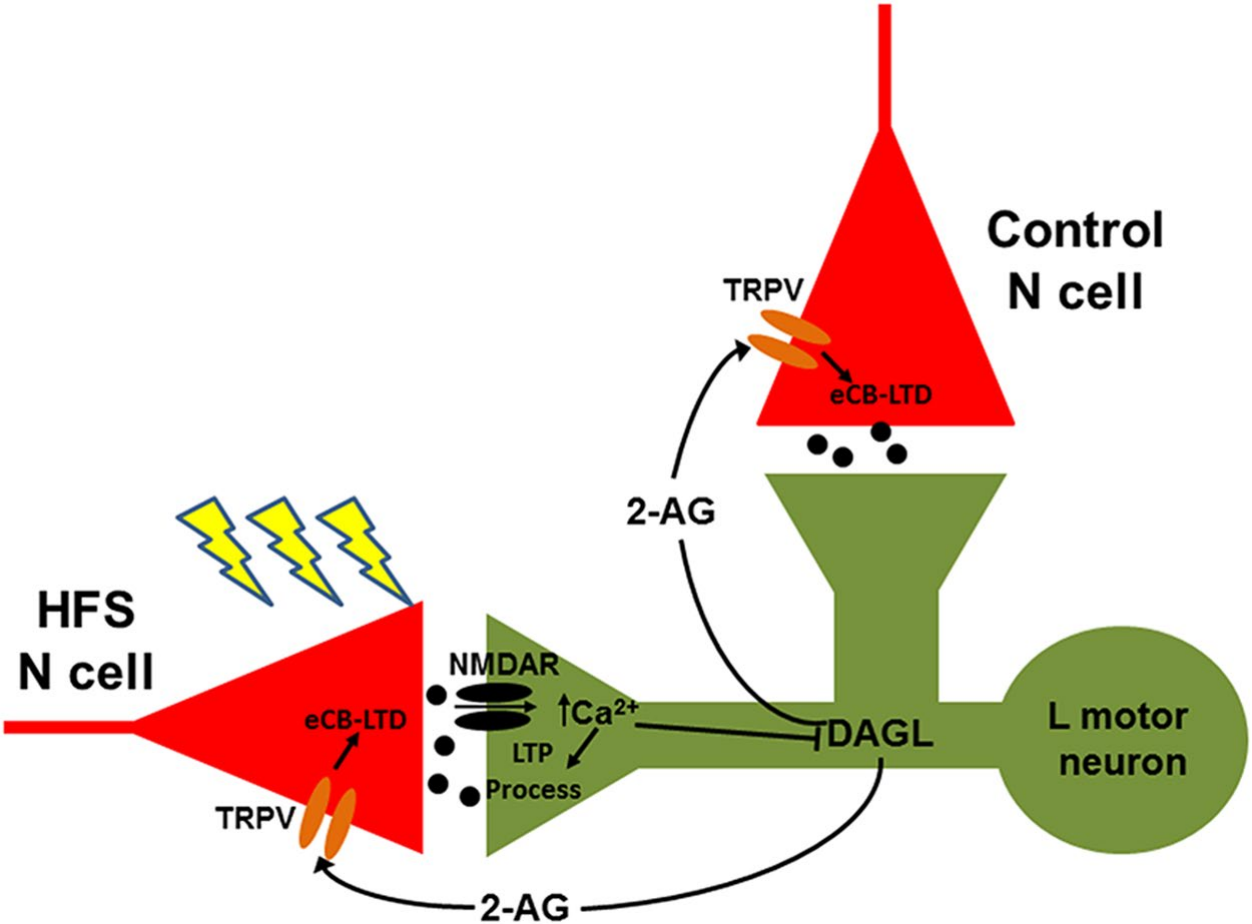
Proposed model of interaction between synaptic modulatory processes. High frequency stimulation (HFS) of an N cell activates postsynaptic NMDARs (in this case in the L motor neuron) that lead to LTP, but only in the activated synapse (i.e. a homosynaptic effect). This LTP is Ca^2+^-dependent, consistent with previous studies of NMDAR-mediated LTP in other *Hirudo* mechanosensory synapses^14,30,31^. This same increase in Ca^2+^ is thought to inhibit DAG lipase (DAGL) –mediated 2-AG synthesis, which can also be initiated by synaptic activity^36,44^. It is not clear if this inhibition of DAGL is due to a direct effect of Ca^2+^ or through Ca^2+^-dependent activation of an intermediary, e.g. a protein kinase. If NMDAR activity is blocked, then 2-AG synthesis is permitted, which in turn elicits eCB-LTD in both the stimulated and control N cell inputs (i.e. a homo- and heterosynaptic effect).

It is surprising that AP5 treatment during N cell HFS resulted not only in blocking LTP, but also uncovering LTD in these activated nociceptive synapses and a potential mechanism for how these two forms of plasticity interact is presented in Fig. 5. This LTD was blocked by inhibitors of 2-AG synthesis and TRPV channel function, similar to the eCB-LTD observed in N cell synapses following LFS of the non-nociceptive T cells^6,36,40^. One feature that both LTP and LTD share is activation of the same postsynaptic cell, the L motor neuron. In the case of eCB-LTD, pharmacological studies have shown that the L motor neuron is the site of 2-AG synthesis^6,40^. Similarly in the present studies, injection of THL into the L motor neuron also blocked the LTD observed in synapses that received HFS in the presence of the NMDAR antagonist AP5. Another similarity was that injection of the TRPV inhibitor SB366791 into the N cell also blocked both LFS-induced LTD and depression observed following HFS in the presence of AP5. In previous studies eCB-LTD produced by LFS was heterosynaptic, with depression observed at both the active and inactive synapses that converge onto a common postsynaptic target (in this case the L motor neuron)^40^. This heterosynaptic feature was also observed in the eCB-LTD observed in the HFS + AP5 conditions. LTD was observed both at the N cell synapse that underwent HFS and by the synapse made by the inactive, contralateral N cell onto the same postsynaptic target. To summarize, the synaptic depression observed when inhibiting NMDARs during HFS is indistinguishable from the eCB-LTD produced by LFS in past experiments.

These results suggest that whether nociceptive synapses undergo NMDAR-LTP or eCB-LTD is determine by the pattern of activity, with LFS producing eCB-LTD and HFS preferentially producing NMDAR-LTP, but also being capable of eliciting eCB-LTD. In previous experiments using LFS, no evidence of LTP was observed when LFS-induced eCB-LTD was blocked using either pre-synaptic THL or postsynaptic SB366791 injections^6,36,44^. This suggests that only HFS has the capacity to engage both NMDAR- and eCB-mediated forms of synaptic plasticity. Based on our BAPTA results, both forms of synaptic plasticity require postsynaptic Ca^2+^ signaling in agreement with earlier studies^30,44^. Nevertheless, the activation of NMDARs not only elicits synaptic potentiation, but also actively inhibits eCB-LTD in these synapses. Exactly what intracellular mechanisms mediated NMDAR-LTP in N cell synapses is unknown at this time, however in *Hirudo* P cell synapses, NMDAR-LTP requires, in addition to increases in intracellular Ca^2+^, activation of protein kinases including CamKII^30,31^. CamKII has been shown to phosphorylate mammalian forms of the 2-AG synthesizing enzymes, DAG lipase, resulting in an inhibition of 2-AG synthesis that did reduce eCB-mediated synaptic depression^54^. It would be interesting to examine not only the potential role of CaMKII, but also whether different levels of Ca^2+^ signaling selectively activate NMDAR-LTP vs. eCB-LTD. However, an important caveat of this potential mechanism is that it is not known if *Hirudo* DAG lipase is similarly regulated by CaMKII. Also studies using other kinases have been shown that they actually increase DAG lipase activity^55^. Alternative mechanisms include regulation of DAG lipase via interactions with Homer or palmitoylation^55^ or regulation at a site independent of DAG lipase, e.g., the 2-AG metabolizing enzyme, MAG lipase. All of these processes imply that HFS-induced inhibition of eCB-LTD is acting at the level of the induction of this form of synaptic depression (a postsynaptic effect). However, it is also possible that inhibition is occurring at the level of expression of synaptic depression (a presynaptic effect). While it is not possible from the current experiments to distinguish between effects of induction vs. expression of depression, the fact that synapses that underwent HFS were still sensitive to exogenous application of 2-AG would seem to suggest that NMDAR-LTP does not alter the ability of the N cells to undergo endocannabinoid-mediated depression.

These findings are compelling because they show an entirely novel mechanism by which activity sufficient to potentiate nociceptive synapses also actively suppresses processes that depress nociceptive synapses. From a functional perspective, these results suggest that injury-induced nociceptive sensitization involves not only strengthening afferent signaling pathways, but also “turning off” modulatory processes that could limit or reverse such sensitization. In the case of endocannabinoid-based modulatory processes this could include stress-mediated analgesia^56,57^ or anti-nociceptive modulation produced by repetitive activation on non-nociceptive afferents^6,58^. This interaction between endogenous pro-nociceptive and anti-nociceptive forms of modulation could also play a role in chronic pain. That is, suppression of endogenous anti-nociceptive forms of modulation may play a critical role in what makes pain pathologically persistent, in parallel with the well-recognized, pro-nociceptive forms of modulation. Under normal conditions, these endogenous, anti-nociceptive forms of modulation may provide a homeostatic role that ultimately ameliorates or even actively reverses the physiological processes that mediate nociceptive sensitization. However, under chronic pain conditions, these corrective factors may be disrupted, potentially allowing pro-nociceptive processes to persist and even expand. Consequently, there is a critical need to understand the cellular mechanisms that mediate this interaction between pro- and anti-nociceptive modulatory processes given that this may lead to novel and effective approaches in treating chronic pain.
